# Barriers, Drivers, and Outcomes in Transitioning Patients With Inflammatory Bowel Disease From Intravenous to Subcutaneous Infliximab

**DOI:** 10.1093/crocol/otaf008

**Published:** 2025-07-20

**Authors:** John R Campion, Emma McCormick, Kate Finn, Aine Keogh, Linda Duane, Rakhi Jose, Laurence J Egan, Eoin Slattery, Mary Hussey

**Affiliations:** Department of Gastroenterology, University Hospital Galway, Galway, Ireland; Department of Gastroenterology, University Hospital Galway, Galway, Ireland; Department of Gastroenterology, University Hospital Galway, Galway, Ireland; Department of Gastroenterology, University Hospital Galway, Galway, Ireland; Department of Gastroenterology, University Hospital Galway, Galway, Ireland; School of Medicine, University of Galway, Galway, Ireland; Department of Gastroenterology, University Hospital Galway, Galway, Ireland; Department of Gastroenterology, University Hospital Galway, Galway, Ireland; School of Medicine, University of Galway, Galway, Ireland; Department of Gastroenterology, University Hospital Galway, Galway, Ireland; School of Medicine, University of Galway, Galway, Ireland; Department of Gastroenterology, University Hospital Galway, Galway, Ireland

**Keywords:** inflammatory bowel disease, biologic therapy, infliximab, subcutaneous

## Abstract

**Background:**

Data are limited on patients’ experience of transition to subcutaneous treatment. This study aimed to determine what factors affect the decision to transition, to assess clinical outcomes and to elucidate patients’ experience of transition.

**Methods:**

This was a longitudinal, observational study carried out at University Hospital Galway, a tertiary referral center in Ireland, from November 2022 to December 2023. The drivers and barriers for patients eligible for transition were measured using a questionnaire with 21, 5-point Likert items. Clinical, biochemical, and patient-reported parameters were assessed at week 8 and week 26. Patients completed a survey at week 26 on their experience of treatment with subcutaneous Infliximab.

**Results:**

Eighty of 144 eligible patients agreed to transition. Treatment persistence was 93.7% at week 26. There was no significant change in mean clinical, biochemical or patient-reported parameters at week 26. In multivariate analysis, there was higher probability of transition among patients with wholly publicly funded healthcare (OR = 3.53, 95% CI, 1.18-11.68). Among those who transitioned, the strongest drivers cited were lifestyle factors while among patients who declined transition, most commonly cited barriers included reduced contact with healthcare professionals. At week 26, 96.1% of respondents reported being able to contact the IBD team when necessary and 87.3% of respondents were satisfied with their monitoring.

**Conclusions:**

Understanding patients’ attitudes toward transition is essential to design a service that meets their needs. Services must be adequately resourced in order to ensure that patients treated with subcutaneous biologics continue to have ready access to high-quality care.

## Graphical Abstract

**Graphical Abstract:**
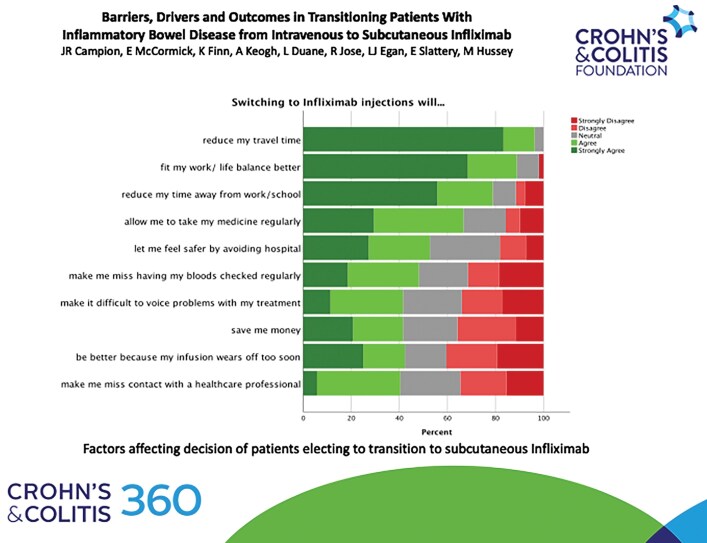

## Introduction

In many health systems internationally, there has been reorientation of services for people with chronic diseases to move their medical care into the community.^1^ This has been driven by a desire to move care closer to the patient, twinned with an economic imperative to rationalize and streamline use and delivery of costly interventions.^2–5^ These drivers have provided impetus to find alternatives to the administration of biologic medications in intravenous (IV) form in a hospital setting.^6,7^ Infliximab (IFX) has been available for several chronic inflammatory indications since 1999, among them induction and maintenance of remission of moderately- to severely active ulcerative colitis (UC) and Crohn’s disease (CD), collectively inflammatory bowel disease (IBD).^8^ Whereas administration of IV IFX requires a patient to attend a hospital infusion unit every 4-8 weeks to receive their drug dose over a period of 30-120 min, subcutaneous (SC) IFX, available since 2020 as biosimilar CT-P13, may be administered by the patient or their carer every 2 weeks, without attending a hospital.^9^ In this context, several potential benefits of using SC IFX compared with the drug’s IV formulation have been identified for patients, clinicians, and health services. These have included patient preference, financial savings, reconfiguration of services for patients with IBD and reduction of infectious risk during the coronavirus (COVID) pandemic.^10–12^

SC IFX has been shown to be noninferior to IV IFX for maintenance of remission of IBD.^13^ It has also been demonstrated that SC IFX has a favorable pharmacokinetic profile, with a steadier drug level between doses.^13,14^ A recent network meta-analysis showed SC IFX had favorable efficacy for maintenance of clinical remission, compared to IV treatment.^15^ Understanding the factors that affect patients’ preferences on the route by which their biologic is administered is an important step in creating an IBD service that meets patients’ needs. Recent research has highlighted that barriers to transition to SC administration may include patients’ concerns about drug efficacy and diminished contact with IBD medical and nursing teams.^10,16^ There is a dearth of research on the potential predictors of patient preference to transition from IV to SC biologic therapy, the effects of transition on patients’ health, social and occupational functioning and the patient experience of transitioning. A deeper understanding of these issues may help gastroenterologists and other members of the healthcare teams treating patients with IBD to ensure that patients are adequately treated and supported when their therapy changes.

## Methods

This was a single-center, longitudinal, observational study carried out at University Hospital Galway between November 2022 and December 2023. Our unit is a publicly funded tertiary referral center for IBD in the West of Ireland, treating patients who live within a 120km radius, and covering urban centers and disparate rural areas. The IBD team includes consultant and trainee gastroenterologists, IBD specialist nurses, infusion nurses, and administrative staff. Patients can contact the IBD team with queries by telephone or email. Infusions of biologic medications are administered at the hospital’s infusion unit.

Ethical approval for the study was granted by the University of Galway Research Ethics Committee and each patient gave informed consent for participation. The study consisted of 3 components; the first studied factors associated with a patient’s decision on the route of administration of the drug, the second examined clinical outcomes after transitioning to SC IFX, and the third studied the patients’ experience of their IBD treatment after transition. The aims of the study were (1) to determine whether there are disease-related factors, infusion-related factors, or economic, social, and personal factors that affected the decision of patients with IBD to transition from IV to SC administration of Infliximab, (2) to examine the clinical outcomes posttransition, and (3) to assess patients’ experience of transitioning to and using SC IFX. Patients were deemed eligible for inclusion in the study if they had UC, CD, or indeterminate colitis, maintained in remission on IV IFX, self-reporting as able to administer SC drug independently or with assistance from a carer, and able to give valid consent to participate. Stable remission was defined as partial Mayo score ≤2, no flare within prior 12 months and recent normal range fecal calprotectin (FCP). Patients fulfilling these criteria were contacted by telephone by the IBD nursing team (advanced nurse practitioner or clinical nurse specialist) and educated on the availability and efficacy of SC IFX, and on the transition pathway from IV to SC administration. The nursing team answered any specific queries posed by the patient. Each patient could decide at the time of initial contact to transition to SC or remain on IV administration. A patient initially deciding to remain on IV IFX could later decide to transition to SC administration. At patient or clinician request, a patient could switch back to IV IFX if a difficulty was identified with the patient’s ability to reliably procure and administer their SC IFX. On the date of their last IFX IV infusion, each patient had laboratory investigations to include full blood count, renal profile, liver profile, C-reactive protein (CRP), IFX trough level, and IFX antidrug antibody level. A stool sample for fecal calprotectin (FCP) was requested from each patient. Each patient received nurse-led education on self-administration of SC IFX. The first dose of SC IFX 120mg was administered in lieu of the patient’s next infusion dose, and administration continued every 2 weeks thereafter. Patients were reminded that they could contact their IBD team with any queries or concerns by telephone, email, or at outpatient clinic.

### Attitudes Toward Route of Administration

All patients who were invited to transition to SC IFX were asked at the initial contact with the IBD nursing team, to complete a questionnaire. This included patients who agreed to transition and those who refused to transition. Those who consented were sent a printed questionnaire by post and, after 2 weeks, those who had not returned the questionnaire by post were offered the opportunity to complete the questionnaire by telephone. The questionnaire consisted of 18 items on the patient’s IBD history, personal and financial circumstances, and 21 items on factors influencing their decision on route of administration. Responses on factors influencing their decision were recorded on a 5-point Likert scale. The distribution of the responses against different questions for the entire dataset was analyzed.

### Factors Associated with Preference on Route of Admission

A bivariable logistic regression model was used to determine which demographic, disease-related, and infusion-related factors were associated with electing to transition. Associations were described by odds ratios and 95% confidence intervals (CI). The significance threshold was set at 5%. These statistical analyses were performed in RStudio (PBC, Boston, MA).

### Clinical Outcomes

Data on patient demographics, disease characteristics and disease activity before the invitation to transition were recorded from the electronic patient record. A database of clinical outcomes, pharmacokinetic and biochemical data, and patient-reported outcome measures (PROMs) was prospectively maintained for the duration of the study. Patients were followed up by the IBD team with phlebotomy and a telephone consultation at 8 weeks and 26 weeks after their first SC dose. Statistical analyses were conducted on an intention-to-treat basis, and those whose treatment changed during the study period were excluded from further analysis.

### Patient Experience of Transitioning to Subcutaneous Dosing

Each patient was contacted by telephone by a doctor or nurse from the IBD team approximately 26 weeks after commencing SC IFX. Patients provided answers to 4 closed questions on a 5-point Likert scale and 2 open questions. An IBD-Control (IBD-C) questionnaire^17^ and a Visual Analogue Scale (VAS) were completed by each patient.

## Results

### Baseline Demographic and Clinical Variables

In total, 144 patients met inclusion criteria and were invited to transition from IV to SC IFX during the study period. Eighty patients (55.5%) agreed to transition to SC IFX and 64 (44.5%) opted to remain on IV IFX. One hundred twenty patients agreed to participate in the study, 75 (62%) switched, and 46 (38%) chose to continue on IV IFX. Table 1 shows the baseline demographic data for all patients. Table 2 shows disease-related variables for each group. IBD diagnosis and demographic factors were evenly distributed between those who agreed to transition and those who remained on IV IFX. Those who switched had a higher baseline IFX trough level at median (IQR) 8.6 (8.7) vs 6.5 (5.2), *P* = .026. Table 3 shows variables related to Infliximab treatment. Those who agreed to switch had a longer journey time to the infusion unit at median (IQR) 60 min (37.5) vs 30 min (45). Respondents who missed more school/work to attend for infusion were more likely to agree to switch to SC administration with median (IQR) 1 (6) days vs 0 (1) days, *P* = .009.

**Table 1.** Distribution of demographic characteristics.

		Overall [*N* = 121]	Switch accepted [*N* = 75]	Switch declined [*N* = 46]	*P*-value^a^
Age (years) [Mean (SD)]		41.7 (14.7)	40.4 (14.7)	43.7 (14.6)	.236
Sex, % (*n*)	Male	62% (75)	61.3% (46)	63% (29)	>.999
	Female	38% (46)	38.7% (29)	37% (17)	
Employment, % (*n*)	Employed	63.9% (62/97)	62.7% (32/51)	65.2% (30)	.379
	Self-Employed	14.4% (14/97)	13.7% (7/51)	15.2% (7)	
	Retired	9.3% (9/97)	7.8% (4/51)	10.9% (5)	
	Student	8.2% (8/97)	11.8% (6/51)	4.3% (2)	
	Other	2.1% (2/97)	3.9% (2/51)		
	Full-Time Parent	2.1% (2/97)		4.3% (2)	
Wholly Publicly Funded Healthcare, % (n)	Yes	58.4% (59/101)	67.3% (37/55)	47.8% (22)	.076
	No	41.6% (42/101)	32.7% (18/55)	52.2% (24)	
Private insurance, % (*n*)	No	55.4% (56/101)	63.6% (35/55)	45.7% (21)	.107
	Yes	44.6% (45/101)	36.4% (20/55)	54.3% (25)	
Drug payment scheme, % (*n*)	No	74.7% (74/99)	70.9% (39/55)	79.5% (35/44)	.453
	Yes	25.3% (25/99)	29.1% (16/55)	20.5% (9/44)	
Relationship status, % (*n*)	Married	49.5% (47/95)	50% (25/50)	48.9% (22/45)	.451
	Single	34.7% (33/95)	32% (16/50)	37.8% (17/45)	
	Relationship	8.4% (8/95)	10% (5/50)	6.7% (3/45)	
	Separated/Divorced	5.3% (5/95)	8% (4/50)	2.2% (1/45)	
	Widowed	2.1% (2/95)		4.4% (2/45)	
Number of dependents, % (*n*)	0	49.5% (46/93)	51% (25/49)	47.7% (21/44)	.989
	1	17.2% (16/93)	16.3% (8/49)	18.2% (8/44)	
	2	12.9% (12/93)	12.2% (6/49)	13.6% (6/44)	
	3+	20.4% (19/93)	20.4% (10/49)	20.5% (9/44)	

^a^Differences between groups for variables summarized as Mean (SD) are assessed using a *t*-test (or the Mann–Whitney test for variables summarized as Median (IQR)); For categorical variables, differences are tested using chi-square (or Fisher’s exact test as appropriate).

**Table 2.** Distribution of disease-related variables.

		Overall [*N* = 121]	Switch accepted [*N* = 75]	Switch declined [*N* = 46]	*P*-value^a^
Disease, % (*n*)	CD	70.2% (85/121)	73.3% (55/75)	66.7% (30/46)	.568
	UC	28.9% (35/121)	26.7% (20/75)	33.3% (15/46)	
Perianal CD	Yes	42.4% (36/85)	72.2% (26/36)	27.8% (10/36)	.073
	No	57.6% (49/85)	53.1% (26/49)	46.9% (23/49)	
Patient’s subjective disease control in past 12 months, % (*n*)	Good Control	89.1% (90/101)	89.1% (49/55)	89.1% (41)	>.999
	Average Control	8.9% (9/101)	9.1% (5/55)	8.7% (4)	
	Poor Control	2% (2/101)	1.8% (1/55)	2.2% (1)	
Other Medications, % (*n*)	Azathioprine	83.3% (20/24)	82.6% (19/23)	100% (1/1)	>.999
	5ASA	8.3% (2/24)	8.7% (2/23)		
	Azathioprine and Vedolizumab	4.2% (1/24)	4.3% (1/23)		
	Methotrexate	4.2% (1/24)	4.3% (1/23)		
PreSwitch HGB [Median (IQR)]		14.3 (2) [*N* = 118]	14.4 (2.2) [*N* = 73]	14.1 (2.3) [*N* = 45]	.316
PreSwitch CRP [Median (IQR)]		1.2 (2.1) [*N* = 118]	1.2 (2.1) [*N* = 73]	1.2 (2) [*N* = 45]	.704
PreSwitch Albumin [Median (IQR)]		44.7 (2.6) [*N* = 118]	44.7 (2.8) [*N* = 73]	44.7 (2.4) [*N* = 45]	.903
PreSwitch IFX level [Median (IQR)]		7.7 (7) [*N* = 118]	8.6 (8.7) [*N* = 73]	6.5 (5.2) [*N* = 45]	.026
PreSwitch IFX ADA, % (*n*)	<10	97.5% (78/80)	98.6% (69/70)	90% (9/10)	.236
	>=10	2.5% (2/80)	1.4% (1/70)	10% (1/10)	
Fecal Calprotectin [Median (IQR)]		35 (80) [*N* = 105]	41 (85) [*N* = 63]	26 (43) [*N* = 42]	.726
Dose (mg/kg), % (*n*)	5	62.7% (74/118)	54.8% (40/73)	75.6% (34/45)	.038
	7	0.8% (1/118)	1.4% (1/73)		
	10	36.4% (43/118)	43.8% (32/73)	24.4% (11/45)	
PreSwitch Mayo Score [Median (IQR)]		0 (1) [*N* = 35]	0.5 (1) [*N* = 18]	0 (2) [*N* = 17]	.971
PreSwitch Harvey Bradshaw Score [Median (IQR)]		1 (2) [*N* = 75]	0 (1.5) [*N* = 47]	1 (2) [*N* = 28]	.667
PreSwitch IBD-C [Median (IQR)]		14 (3.8) [*N* = 118]	14 (4) [*N* = 73]	14 (2) [*N* = 45]	.525
PreSwitch VAS [Median (IQR)]		90 (15) [*N* = 118]	85 (20) [*N* = 73]	90 (15) [*N* = 45]	.263

^a^Differences between groups for variables summarized as Mean(SD) are assessed using a *t*-test (or the Mann–Whitney test for variables summarized as Median (IQR)); For categorical variables, differences are tested using chi-square (or Fisher’s exact test as appropriate).

**Table 3.** Distribution of infusion-related variables.

		Overall [*N* = 121]	Switch accepted [*N* = 75]	Switch declined [*N* = 46]	*P*-value^a^
Duration of treatment (years) [Median (IQR)]		8 (8.2) [*N* = 92]	7 (8.2) [*N* = 48]	8 (8.2) [*N* = 44]	.541
Interval in weeks [Median (IQR)]		8 (2) [*N* = 117]	8 (2) [*N* = 72]	8 (0) [*N* = 45]	.017
Travel time (min) [Median (IQR)]		45 (40) [*N* = 96]	60 (37.5) [*N* = 51]	30 (45) [*N* = 45]	.003
Time in unit (min) [Median (IQR)]		120 (30) [*N* = 97]	120 (22.5) [*N* = 51]	120 (30)	.211
Miss school/work, % (n)	No	52% (52/100)	42.6% (23/54)	63% (29)	.066
	Yes	48% (48/100)	57.4% (31/54)	37% (17)	
How many days missed of school/work [Median (IQR)]		0 (5.2) [*N* = 96]	1 (6) [*N* = 53]	0 (1) [*N* = 43]	.009

^a^Differences between groups for variables summarized as Mean(SD) are assessed using a *t*-test (or the Mann–Whitney test for variables summarized as Median (IQR)); For categorical variables, differences are tested using chi-square (or Fisher’s exact test as appropriate).

Binary logistic regression models were constructed for demographic, disease-related, and infusion-related variables to predict the probability of agreeing to transition to SC IFX. Results of these analyses are shown in Supplementary Tables 5, 6, and 7, respectively. In univariate analysis of demographic factors, variables associated with probability of switching were whether school/work was missed to attend for infusion, number of days of school/work missed to attend for infusion, frequency of infusions, travel time to infusion unit. Missing school/work to attend for infusion was associated with higher chances of switching to SC IFX (OR = 2.30, 95% CI, 1.04-5.22). The number of days missed of school/work was associated with higher chances as the number of days increase (OR = 1.23, 95% CI, 1.07-1.45). A longer interval between infusions was negatively associated with switching (OR = 0.68, 95% CI, 0.49-0.91). Travel time was associated with higher chances as the travel time increases (OR = 1.01, 95% CI, 1.00-1.03). Patients with wholly publicly funded healthcare had higher chances of switching (OR = 2.24, 95% CI, 1.01-5.09). In univariate analysis the only disease-related variable associated with probability of switching was IFX trough level, with higher chances as the IFX trough level increase (OR = 1.10, 95% CI, 1.02-1.19).

In multivariate analysis (Table 4), only wholly publicly funded healthcare remained significant with higher chances to switch for those with wholly publicly funded healthcare (OR = 3.53, 95% CI 1.18 to 11.68).

**Table 4.** Multiple binary logistic regression model.

			95% confidence interval		
Terms	Contrast	OR	Lower bound	Upper bound	*P*-value
Wholly Publicly Funded Healthcare	Yes vs No	3.53	1.18	11.68	.029
Infusion interval		0.75	0.47	1.15	.203
Travel time		1.01	1.00	1.03	.071
Miss school/work	Yes vs No	1.75	0.31	10.30	.522
Number of days of school/work missed		1.12	0.83	1.54	.452
PreSwitch IFX level		1.09	0.97	1.23	.156
Dose (mg/kg)	5 vs 10	0.75	0.22	2.53	.641

### Clinical Course After Transition to Subcutaneous Infliximab

**Figure 1.**
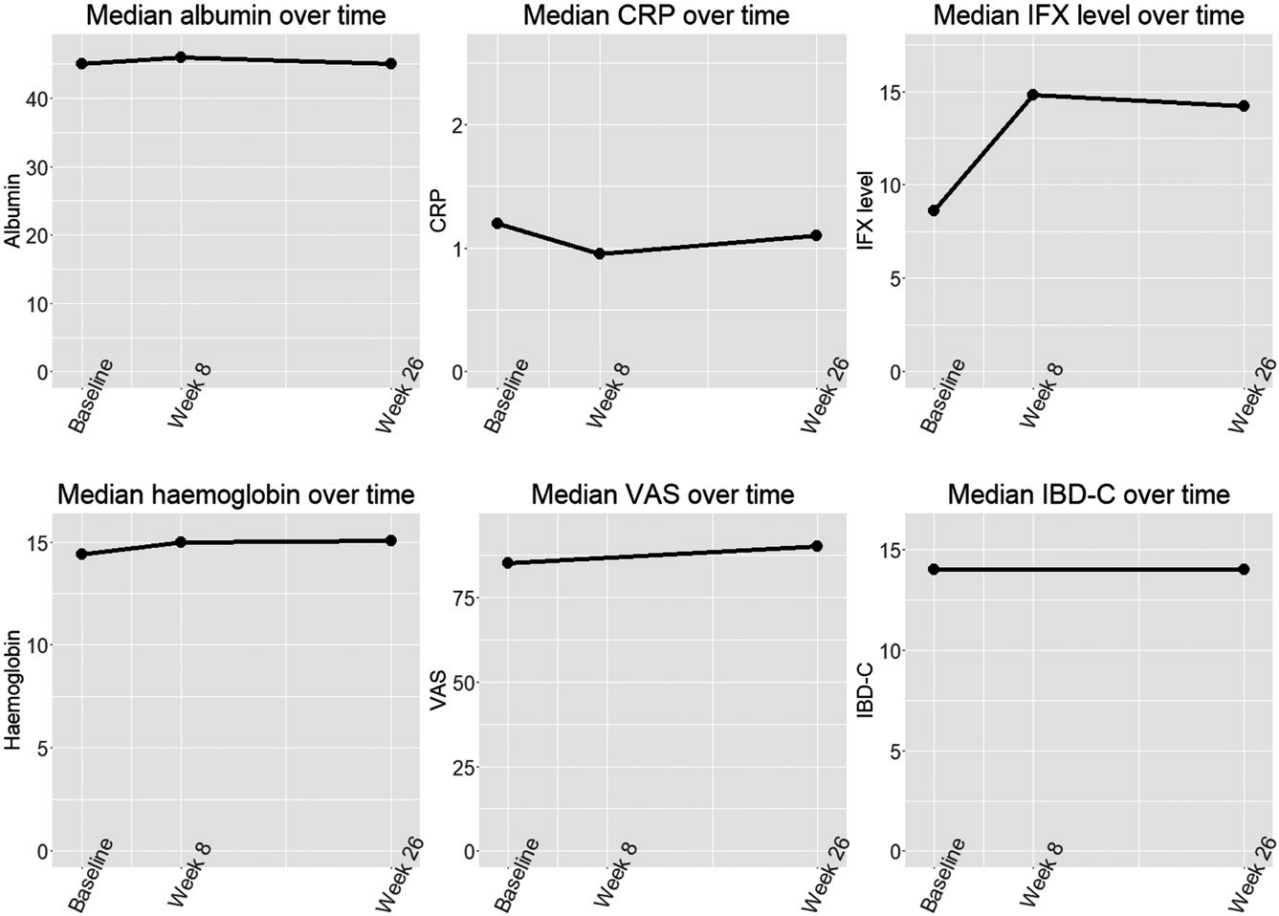
Trajectory of biochemical and patient-reported measures of disease activity at baseline, 8 weeks and 26 weeks.

For patients who switched to SC IFX, clinical, and biochemical parameters were recorded again at week 8 and week 26 after transition to SC IFX. Figure 1 shows trajectory of biochemical and patient-reported measures of disease activity from baseline through week 8 to week 26. Albumin levels increased significantly between preswitch and week 8 for those who switched (mean increase of 1.2 g/L, 95% CI, 0.64-1.85). IFX trough levels increased significantly between preswitch and week 8 for those who switched (mean increase of 5.93 mg/mL, 95% CI, 3.91-7.95). Hemoglobin levels increased significantly between preswitch and week 8 for those who switched (mean increase of 0.48 g/dL, 95% CI, 0.19-0.78). At week 26, only hemoglobin showed a significant increase from preswitch for those who switched (mean increase of 0.49 g/dL, 95% CI, 0.13-0.86). This increase is similar to that detected at week 8 indicating that hemoglobin levels remained approximately constant between week 8 and week 26. Patient-reported outcome measures IBD-C and VAS were recorded before switching and 26 weeks after switching. IBD-C increased by mean 1.52 points, 95% CI, −1.68 to 4.72 and VAS increased by mean 1.46 points, 95% CI, −4.14 to 7.07. Twenty-six weeks after switching to SC IFX, *N* = 5 patients (6.3%) had discontinued SC IFX. One patient was switched to Upadacitinib, due to secondary loss of response with good drug levels, while 4 patients (5%) had reverted to IV IFX. Of those who had reverted to treatment with IV IFX, one patient requested switch back as he felt his health had been generally better on IV IFX, though there was no objective deterioration in his clinical condition or his laboratory indices. One patient self-discontinued SC treatment without consulting the IBD team and subsequently had a deterioration in symptoms requiring reinduction with IV IFX. One patient had difficulty self-administering his SC medication, and one patient was switched back to IV treatment as she had worsening of her perianal Crohn’s disease. There were no other significant adverse events reported.

### Attitudinal Survey

**Figure 2.**
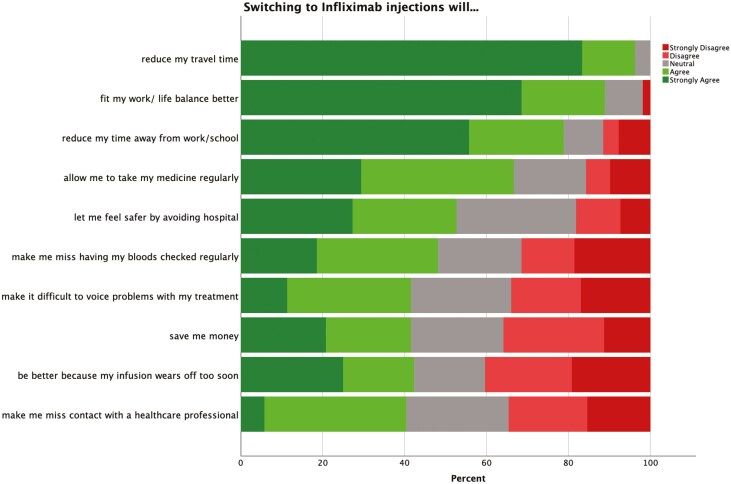
Factors affecting decision of patients electing to transition to SC IFX.

**Figure 3.**
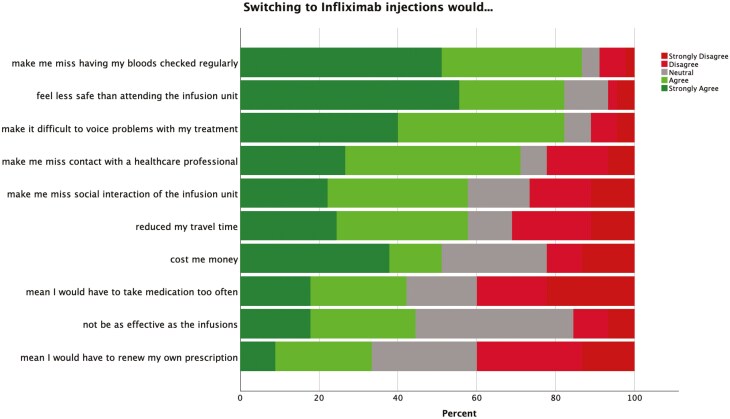
Factors affecting decision of patients electing to remain on IV IFX.

All patients who had been offered the opportunity to switch to SC IFX were invited to participate in the questionnaire study. One hundred one patients (60 male, 41 female) agreed to participate in the survey and returned the questionnaire, giving a response rate of 70.1%. Sixty-eight patients (67.3%) had Crohn’s disease and 33 (32.7%) had UC. Participants’ ages ranged from 18 to 82 (median 41) years. Responses to the attitudinal Likert items with the strongest agreement for patients who chose SC IFX and patients who chose IV IFX are shown in Figure 2 and Figure 3, respectively. Among respondents who elected to transition, the strongest factors (% agree/strongly agree on 5-point Likert scale) were, “Switching to injections will…” (1) reduce my travel time (94.5%), (2) fit my work/life balance better (87.3%), and (3) reduce my time away from work/school (74.5%). Among respondents who opted to remain on IV IFX, the strongest factors (% agree/strongly agree) were, “Switching to injections would…” (1) make me miss having my bloods checked regularly (84.8%), (2) feel less safe than attending the infusion unit (80.4%), (3) make it difficult to voice concerns about my treatment (80.4%) and (4) make me miss regular contact with a healthcare professional (69.6%).

### Patient Experience of Transition

Of those who switched to SC IFX, 52 patients (69.3%) agreed to complete the post-transition questionnaire. 84.6% of respondents agreed/strongly agreed with the statement, “I am satisfied with IFX injections,” 86.5% of respondents agreed/strongly agreed with the statement, “My IBD is well-controlled with IFX injections,” 96.1% of respondents agreed/strongly agreed with the statement, “I am able to contact my IBD team if I need to” and 87.3% of respondents agreed/strongly agreed with the statement, “I am satisfied with how my IBD has been monitored since switching to IFX injections.”

## Discussion

### Clinical Outcomes

This study provides further evidence that SC IFX is safe, effective, and well-tolerated by those maintained in remission on IV IFX, who choose to transition to the SC formulation of the drug. Indices of inflammatory burden and PROMs remained broadly stable. These findings concur with prior studies on the safety and efficacy of transitioning to SC IFX.^11,18^ Treatment persistence at week 26 was high and there was a high level of patient satisfaction with the transition process and with their monitoring and treatment after transition.

### Patient-Borne Costs of IBD

In Ireland, medications, primary care, and hospital care are provided free at the point of access to those whose income falls under a certain threshold. Those whose income is above that threshold must pay part of the cost when they avail of health services, with the personal cost for medication from a community pharmacy capped at €80 per household per month by registering for the Drug Payment Scheme.^19^ Private health insurance is optional and policies typically cover the cost of attending an infusion unit, but not the cost of medication dispensed from a community pharmacy. Until recently, the cost for attending an infusion unit for patients with wholly publicly funded healthcare was €80 per session, but in April 2023, the fee for attending an infusion unit was abolished, making infusions free at the point of access for all patients. Data from the European Integrated Price Information Database (EURIPID) puts the average cost of IV IFX for a 70 kg patient receiving 5 mg/kg every 8 weeks at €1123, compared to €1440 for 4 doses of SC IFX during an 8 week period.^20^

Results from our survey on patient attitudes to route of administration depict the many and varying influences on each patient’s decision on route of biologic administration. Prior research has highlighted the high financial patient costs of IBD.^21^ Results from this study also confirm the other complex, personal costs associated with a diagnosis of IBD outlined by previous studies.^22^ In making decisions about their IBD care, patients balance impacts of the social, financial, and health-related costs of the disease. The impact of each of these cost types is different for each patient, depending on their personal, professional, and financial circumstances. For some patients, attending hospital regularly for IV infusions can have a significant, negative effect on their life by interrupting their employment, education, and family life, making SC administration an attractive option. Among those patients who decided to transition to SC IFX, the greatest influences are related to lessening the impact of IBD on their ability to participate in society as those without IBD are able to do. This corroborates earlier work on the financial and social toxicity of IBD.^23^ SC IFX may therefore provide an effective means to reduce morbidity of IBD for these patients.^12^

Prior research had suggested that a shorter duration of treatment with IV IFX was associated with increased willingness to switch to SC IFX.^10^ This association is not endorsed in our cohort. Similar to prior research on patient choice in IBD treatment, among patients who elected to remain on IV IFX, the principal drivers related to fears around access to healthcare and safety of disease monitoring in the absence of regular attendance at an infusion unit.^11,16^ Reassuringly, those fears were not borne out by the posttransition survey, where most respondents had a positive experience of the transition process and the availability of their IBD team’s clinical expertise when they were in need of it. Financial cost is a significant barrier to transition for those IBD patients who must pay for medication dispensed in the community, despite SC administration of biologic medicines accruing financial savings to public health system and insurers, when compared to IV administration.^12^ Understanding patients’ attitudes toward SC self-administration and the factors influencing the decision to transition allows clinicians to co-design IBD services that meet their patients’ needs. It is important that health service planners and policymakers account for the effect of redistribution of costs to patients, in any new policies.

### Roles of the IBD Team and Other Patients with IBD

To support patients who choose SC biologic administration, IBD medical and nursing teams must be adequately resourced, in order to ensure that these patients continue to have ready access to high-quality care. The role of the IBD nursing team in patient education is highlighted in these results by the high level of patient knowledge on efficacy of SC IFX; concerns about effectiveness of the SC formulation did not rank highly among those choosing to remain on IV IFX. These data concur with prior studies, which showed that meeting with a healthcare professional increases the likelihood a patient will elect to transition to SC administration of their biologic treatment.^24^ The higher preswitch IFX trough level among those who switched may be reflective of reluctance among those with lower levels to alter their treatment regimen, in case this would lead to a deterioration in disease control. The importance of the IBD nursing role is also highlighted by the desire expressed by the entire cohort to continue to have access to the IBD team. The nursing staff at the infusion unit are an important resource for IBD patients to discuss their disease and treatment. Assurance that they will have ready access to the clinical team provides a sense of safety to patients with IBD. The patients surveyed indicated that they value laboratory testing in monitoring of their disease. Laboratory monitoring and exposure to clinical staff and peers with IBD provide a sense of safety and confidence in control of their disease. Among those who opted to remain on IV IFX, 58% cited a loss of social interaction as a factor influencing their decision. This finding may highlight the role of IBD patients as peer educators and supporters to their fellow IBD patients. In many cases, a person’s only regular interaction with another person with IBD may be when they attend the infusion unit. This also suggests a potential use of peer educators and peer support groups in facilitating transition from IV to SC biologic administration.

The strengths of this study include the large number of patients enrolled, short interval between the offer to transition and administration of the attitudinal questionnaire, and the maintenance of a prospective database of clinical, biochemical, and patient-reported outcomes. Limitations of the study include its single-center design and relatively short follow-up period for clinical outcomes.

This study provides insights on patient choices in a single healthcare system. It is likely that the financial structures of different countries’ healthcare systems will play an important role in influencing patients’ decisions on the route of administration of advanced therapies. Those healthcare systems that can provide adequate community-based monitoring and treatment are likely to see higher levels of uptake of SC biologics, whereas healthcare systems where patients are not confident of safe and effective community-based care may struggle to transition patients to SC treatment. In some health systems, there may be an incentive for hospitals to continue treating patients with IV therapy, in order to receive additional activity-based funding.^25^

Further study is needed on the impact of SC transition on healthcare-seeking behavior of patients. It would also be beneficial for future research to investigate strategies to increase patients’ active involvement in management of their IBD.

## Supplementary Material

otaf008_suppl_Supplementary_Materials_1

## Data Availability

Study data is available from the corresponding author on reasonable request.

## Notes

Conference Presentation: Part of this work presented at UEG Week, Copenhagen, 2023.

## Abbreviations

ADA: anti-drug antibodies
CD: Crohn’s disease
CI: confidence interval
CRP: C-reactive protein
COVID: coronavirus disease
DPS: drug payment scheme
EPR: electronic patient record
FBC: full blood count
FCP: faecal calprotectin
IFX: infliximab
IBD: inflammatory bowel disease
IBD-C: inflammatory bowel disease control questionnaire
IQR: interquartile range
IV: intravenous
OR: odds ratio
PROM: patient-reported outcome measure
SC: subcutaneous
UC: ulcerative colitis
VAS: visual analogue scale
