# Supplementary material for: Barriers, Drivers, and Outcomes in Transitioning Patients With Inflammatory Bowel Disease From Intravenous to Subcutaneous Infliximab
Source: Crohns Colitis 360. 2025 Jul 20;7(3):otaf008. doi: 10.1093/crocol/otaf008 (PMC12287920; doi:10.1093/crocol/otaf008)
Supplement: otaf008_suppl_Supplementary_Materials_1 [file otaf008_suppl_supplementary_materials_1.pdf]

Supplementary Material

Table 5. Univariate analysis of demographic variables

			(95% confidence interval		
**Terms**	**Contrast**	**OR**	**Lower bound**	**Upper bound**	**Pvalue**
Age (years)		0,98	0,96	1,01	0.235
Sex	Male vs Female	0,93	0,43	1,98	0.851
Employment	Other vs Employed	1,87	0,53	7,62	0.343
	Retired vs Employed	0,75	0,17	3,09	0.688
	Self-Employed vs Employed	0,94	0,29	3,05	0.913
Wholly Publicly-Funded Healthcare	Yes vs No	2,24	1,01	5,09	0.05
Private insurance	Yes vs No	0,48	0,21	1,06	0.072
Drug payment scheme	Yes vs No	1,60	0,64	4,20	0.328
Relationship status	Married vs Single	1,21	0,49	2,96	0.679
	Relationship vs Single	1,77	0,37	9,80	0.48
	Separated/Divorced/Widowed vs Single	1,42	0,27	8,15	0.678
Number of dependents	1 vs 0	0,84	0,27	2,66	0.764
	2 vs 0	0,84	0,23	3,06	0.788
	3+ vs 0	0,93	0,32	2,76	0.9

Table 6. Univariate analysis of disease related variables

			(95% confidence interval		
**Terms**	**Contrast**	**OR**	**Lower bound**	**Upper bound**	**Pvalue**
Disease	UC vs CD	0,73	0,33	1,64	0.437
Disease control	Good Control vs Average Control	0,96	0,22	3,84	0.949
	Poor Control vs Average Control	0,80	0,03	24,81	0.887
Pre-Switch HGB		1,15	0,93	1,45	0.207
Pre-Switch CRP		1,03	0,98	1,15	0.508
Pre-Switch Albumin		1,01	0,87	1,16	0.905
Pre-Switch IFX level		1,10	1,02	1,19	0.021
Pre-Switch IFX ADA	>=10 vs <10	0,13	0,00	3,49	0.162
Faecal Calprotectin		1,00	1,00	1,01	0.29
Dose (mg/kg)	5 vs 10	0,40	0,17	0,90	0.031
Pre-Switch Mayo Score		0,80	0,44	1,37	0.436
Pre-Switch Harvey Bradshaw Score		0,85	0,63	1,07	0.225
Pre-Switch IBD-C		1,00	0,92	1,09	0.982
Pre-Switch VAS		0,98	0,95	1,01	0.196

Table 7. Univariate analysis of infusion-related variables

			(95% confidence interval		
**Terms**	**Contrast**	**OR**	**Lower bound**	**Upper bound**	**Pvalue**
Duration of treatment		0,98	0,92	1,05	0.542
Interval in weeks		0,65	0,45	0,89	0.013
Travel time		1,01	1,00	1,03	0.024
Time in unit		1,01	1,00	1,03	0.119
Miss school/work	Yes vs No	2,30	1,04	5,22	0.043
How many days missed of school/work		1,23	1,07	1,45	0.008

Questionnaire to assess patient preferences in relation to subcutaneous vs. intravenous Infliximab

Patient Details

Initials: ☐ **Age:** ☐ **Gender:** Male: ☐ Female ☐

Disease: Crohns Disease ☐ Ulcerative Colitis ☐ Unsure ☐

In the past 12 months, I feel my disease is:

Well-controlled ☐ Average ☐ Poorly controlled ☐

Frequency of Infusions: 8 weekly ☐ 6 weekly ☐ 4 weekly ☐

Employment: Employed ☐ Self-employed ☐ Retired ☐

Student ☐ Carer ☐ Full-time Parent ☐

Other: ______________________________

Medical Card holder: Yes ☐ No ☐

Medical Insurance: Yes ☐ No ☐

Drug Payment Scheme: Yes ☐ No ☐

Relationship status? ☐

Number of dependents? ☐

Where applicable do you pay a fee for attending for your Infusion: i.e. Chair cost? Yes ☐ No ☐

If so how much? ☐

How often? ☐

How long does it take you to travel to receive your infusion?

☐

How long on average do you spend per infusion at the infusion unit?

☐

Do you miss work/school as a result of your infusion? Yes ☐ No ☐

If so how many days per calendar year (estimate) ☐

Would you like to switch to an injectable version of your medicine? ***Yes*** ☐ ***No*** ☐

Questionnaire to assess patient preferences in relation to subcutaneous vs. intravenous Infliximab

Which of the following would influence your decision to switch to an injectable form of Infliximab at home? *Please circle your answer*

1. **I'm afraid of injections**

   (a) Strongly agree **(b)** Somewhat agree **(c)** Neither agree nor disagree

   (d) Somewhat disagree **(e)** strongly disagree

2. **The frequency of injections is too much**

   (a) Strongly agree **(b)** Somewhat agree **(c)** Neither agree nor disagree

   (d) Somewhat disagree **(e)** strongly disagree

3. **I would miss regular contact with a Health care Professional.**

   (a) Strongly agree **(b)** Somewhat agree **(c)** Neither agree nor disagree

   (d) Somewhat disagree **(e)** strongly disagree

4. **I would miss having my bloods checked regularly.**

   (a) Strongly agree **(b)** Somewhat agree **(c)** Neither agree nor disagree

   (d) Somewhat disagree **(e)** strongly disagree

5. **I would miss the social interaction of attending the infusion unit.**

   (a) Strongly agree **(b)** Somewhat agree **(c)** Neither agree nor disagree

   (d) Somewhat disagree **(e)** strongly disagree

6. **I feel safer attending the infusion unit**

   (a) Strongly agree **(b)** Somewhat agree **(c)** Neither agree nor disagree

   (d) Somewhat disagree **(e)** strongly disagree

7. **I would anticipate that switching to the injection would cost me more money**

   (a) Strongly agree **(b)** Somewhat agree **(c)** Neither agree nor disagree

   (d) Somewhat disagree **(e)** strongly disagree

8. I would anticipate that switching to the injection would save me money

(a) Strongly agree **(b)** Somewhat agree **(c)** Neither agree nor disagree

(d) Somewhat disagree **(e)** strongly disagree

9. Switching to injections would fit my work/life balance better

(a) Strongly agree **(b)** Somewhat agree **(c)** Neither agree nor disagree

(d) Somewhat disagree **(e)** strongly disagree

10. Switching to injections would reduce my travel time.

(a) Strongly agree **(b)** Somewhat agree **(c)** Neither agree nor disagree

(d) Somewhat disagree **(e)** strongly disagree

11. Switching to injections would reduce my childcare costs.

(a) Strongly agree **(b)** Somewhat agree **(c)** Neither agree nor disagree

(d) Somewhat disagree **(e)** strongly disagree

12. Switching to injections would reduce time away from work/school.

(a) Strongly agree **(b)** Somewhat agree **(c)** Neither agree nor disagree

(d) Somewhat disagree **(e)** strongly disagree

13. I would feel safer avoiding the hospitals by taking my medicine in my own home

(a) Strongly agree **(b)** Somewhat agree **(c)** Neither agree nor disagree

(d) Somewhat disagree **(e)** strongly disagree

14. I do not want to be responsible for collecting my medicine.

(a) Strongly agree **(b)** Somewhat agree **(c)** Neither agree nor disagree

(d) Somewhat disagree **(e)** strongly disagree

15. I do not want to be responsible for storing my medication.

(a) Strongly agree **(b)** Somewhat agree **(c)** Neither agree nor disagree

(d) Somewhat disagree **(e)** strongly disagree

16. I do not want to have to renew my prescription.

(a) Strongly agree **(b)** somewhat agree **(c)** Neither agree nor disagree

(d) Somewhat disagree **(e)** strongly disagree

17. I do not think the injection will be as effective.

(a) Strongly agree **(b)** Somewhat agree **(c)** Neither agree nor disagree

(d) Somewhat disagree **(e)** strongly disagree

18. I will have to take my medicine more regularly.

(a) Strongly agree **(b)** somewhat agree **(c)** Neither agree nor disagree

(d) Somewhat disagree **(e)** strongly disagree

19. The injection may have more side effects.

(a) Strongly agree **(b)** Somewhat agree **(c)** Neither agree nor disagree

(d) Somewhat disagree **(e)** strongly disagree

20. I will not be able to voice my problems with my treatments as easily.

(a) Strongly agree **(b)** Somewhat agree **(c)** Neither agree nor disagree

(d) Somewhat disagree **(e)** strongly disagree

21. I want to switch to injections as my infusion wears off before the next one is due.

(a) Strongly agree **(b)** Somewhat agree **(c)** Neither agree nor disagree

(d) Somewhat disagree **(e)** strongly disagree

22. Someone I know takes injections for a medical condition. Yes☐ No☐

23. Do you have any other thoughts on switching to injections?

____

____

____
